# Antidepressant use during pregnancy and risk of autism spectrum disorder and attention deficit hyperactivity disorder: systematic review of observational studies and methodological considerations

**DOI:** 10.1186/s12916-017-0993-3

**Published:** 2018-01-15

**Authors:** Daniel R. Morales, Jim Slattery, Stephen Evans, Xavier Kurz

**Affiliations:** 1 Pharmacovigilance and Epidemiology Department, European Medicines Agency, 30 Churchill Place, Canary Wharf, London, E14 5EU UK; 2 Division of Population Health Sciences, University of Dundee, Dundee, UK; 3 Department of Medical Statistics, London School of Hygiene and Tropical Medicine, University of London, London, UK

**Keywords:** Autism, Attention Deficit Hyperactivity Disorder, Pregnancy, Antidepressant, Meta-analysis, Epidemiology

## Abstract

**Background:**

Antidepressant exposure during pregnancy has been associated with an increased risk of autism spectrum disorder (ASD) and attention deficit hyperactivity disorder (ADHD) in several observational studies. We performed a systematic review of these studies to highlight the effect that important methodological limitations have on such analyses and to consider approaches to the conduct, reporting and interpretation of future studies.

**Methods:**

A review of MEDLINE and EMBASE identified case–control, cohort and sibling studies assessing the risk of ASD and ADHD with antidepressant use during pregnancy. Approaches to confounding adjustment were described. Crude and adjusted effect estimates for comparisons between antidepressant exposure during pregnancy vs. all unexposed women were first meta-analysed using a generic inverse variance method of analysis, followed by effect estimates for alternative pre-selected comparison groups.

**Results:**

A total of 15 studies measuring ASD as an outcome (involving 3,585,686 children and 40,585 cases) and seven studies measuring ADHD as an outcome (involving 2,765,723 patients and 52,313 cases) were identified. Variation in confounding adjustment existed between studies. Updated effect estimates for the association between maternal antidepressant exposure during pregnancy vs. all unexposed women remained statistically significant for ASD (adjusted random-effects risk ratio [RaRR] 1.53, 95% confidence interval [CI] 1.31–1.78). Similar significant associations were observed using pre-pregnancy maternal antidepressant exposure (RaRR 1.48, 95% CI 1.29–1.71) and paternal antidepressant exposure during pregnancy (1.29, 95% CI 1.08–1.53), but analyses restricted to using women with a history of affective disorder (1.18, 95% CI 0.91–1.52) and sibling studies (0.96, 95% CI 0.65–1.42) were not statistically significant. Corresponding associations for risk of ADHD with exposure were: RaRR 1.38, 95% CI 1.13–1.69 (during pregnancy), RaRR 1.38, 95% CI 1.14–1.69 (during pre-pregnancy), RaRR 1.71, 95% CI 1.31–2.23 (paternal exposure), RaRR 0.98, 95% CI 0.77–1.24 (women with a history of affective disorder) and RaRR 0.88, 95% CI 0.70–1.11 (sibling studies).

**Conclusions:**

Existing observational studies measuring the risk of ASD and ADHD with antidepressant exposure are heterogeneous in their design. Classical comparisons between exposed and unexposed women during pregnancy are at high risk of residual confounding. Alternative comparisons and sibling designs may aid the interpretation of causality and their utility requires further evaluation, including understanding potential limitations of undertaking meta-analyses with such data.

**Electronic supplementary material:**

The online version of this article (doi:10.1186/s12916-017-0993-3) contains supplementary material, which is available to authorized users.

## Background

The safety of medicines during pregnancy and their effect on foetal development is an important concern for public health and medicines regulatory agencies worldwide. Clinical drug safety trials in pregnant women are usually infeasible or unethical to perform. Therefore, evidence from observational studies is relied upon to evaluate the safety of medicines used during pregnancy to inform regulatory decision-making and assess the need for risk-minimisation measures.

Childhood neurodevelopmental disorders are a group of conditions with onset in the developmental period, often before children reach school age. In the *Diagnostic and Statistical Manual of Mental Disorders 5* (DSM-5), neurodevelopmental disorders are organised into seven subcategories: intellectual disabilities, communication disorders, autism spectrum disorder (ASD), attention deficit hyperactivity disorder (ADHD), specific learning disorders, motor disorders and other neurodevelopmental disorders (those disorders having symptoms consistent with, but which fail to meet full diagnostic criteria for, any disorder in the neurodevelopmental disorder diagnostic class) [1]. Potential risk factors for neurodevelopmental disorders include male sex, genetic influences, health behaviours associated with socioeconomic characteristics (such as abuse of alcohol or use of recreational drugs), parental mental health disorders, toxin exposure, obesity, and prenatal and delivery complications [2–4]. However, these disorders often encompass a spectrum of presentations, and there are likely to be potentially unknown environmental or genetic risk factors. Partly because of this spectrum, the definition will depend on the age at assessment and it is very difficult to standardise within and between studies. It is certainly not a simple ‘yes/no’ binary outcome.

The incidence of neurodevelopmental disorders such as ASD and ADHD is reported to have increased in recent decades, the reasons for which are uncertain [2, 5]. This may in part be related to changing diagnostic and coding practices but the effects of maternal exposure to medicines during pregnancy has also been proposed [6]. In Europe, between 3% and 17% of women reportedly experience depression during pregnancy [7–10] and antidepressants are one of the most commonly used medicines in pregnancy [11–13]. In a study of selective serotonin reuptake inhibitor (SSRI) exposure before, during and after pregnancy in six European regions, the prevalence of SSRI exposure at any point during pregnancy ranged between 1.2% and 4.5% [14]. This was highest in the UK and increased between 2004 and 2010.

Observational studies evaluating the safety of antidepressant exposure during pregnancy face important challenges. These include: confounding by indication (where the indication for exposure is directly linked to the risk of health outcomes), difficulties in adjusting for the severity of disease (women with severe symptoms are more likely to be prescribed a medicine during pregnancy where the perceived benefit of treatment may be greater than those with less severe symptoms), assessing antidepressant exposure as a class effect when recommendations for the choice of antidepressants may vary between countries [14] and differences in data sources in terms of completeness and accuracy of information on exposure, outcome, confounders and family history of relevant genetic disorders. Studies using alternative antidepressants as the comparator may be less subject to confounding than those which use unexposed mothers.

Several observational studies have investigated the association between maternal antidepressant exposure during pregnancy and the risk of neurodevelopmental outcomes, which has resulted in the first meta-analysis of observational studies by Man et al. describing a significantly increased risk of ASD in children exposed in utero to SSRIs [15]. Some observational studies included in that report contained a number of important limitations inherent in this type of research, including those described above that raise questions as to the value of a meta-analytical approach. Furthermore, additional observational studies have since emerged that may cause significant public concern [16–19].

In September 2016, the Pharmacovigilance Risk Assessment Committee (PRAC) of the European Medicines Agency (EMA) concluded that the current evidence does not support a causal association and that the available studies on the risk of ASD after in utero exposure to SSRIs are conflicting, in part due to the different study designs and study populations chosen for analysis [20]. The aim of this study was to perform a systematic review of observational studies examining the risk of ASD and ADHD associated with maternal antidepressant exposure during pregnancy and, supporting this, to highlight their methodological limitations and to consider approaches to the conduct, reporting and interpretation of studies investigating antidepressant exposure during pregnancy and risk of neurodevelopmental outcomes. The limitations of trying to obtain a single answer using meta-analysis are also considered. This article does not discuss the conclusion of the PRAC.

## Methods

A systematic review of MEDLINE and EMBASE was performed using a pre-specified search strategy to identify all case–control, cohort or sibling studies published on or before May 2017 evaluating the risk of ASD and ADHD following antidepressant exposure during pregnancy. The search strategy consisted of the following search terms: (SSRI* OR serotonin uptake inhibitor OR antidepressant* OR fluoxetine OR citalopram OR escitalopram OR paroxetine OR sertraline OR venlafaxine OR trazodone OR mirtazapine OR duloxetine OR amitriptyline OR nortriptyline OR imipramine OR fluvoxamine OR nefazadone) AND (pregnancy OR pregnant OR prenatal) AND (autism OR autistic OR pervasive developmental disorder OR ASD OR ADHD OR attention deficit). Titles and abstracts were screened and full texts of relevant articles assessed for eligibility. Only English-language publications and published data were included. A cumulative review of available data (for example, pharmacoepidemiological studies and the published literature) submitted to the EMA by marketing authorisation holders of all SSRI drugs following a request by the PRAC in 2016 was screened to identify additional studies. Methodological quality and risk of bias for the main comparison between exposed vs. unexposed women during pregnancy were evaluated for each study using the ROBINS-I tool, including misclassification of exposure, misclassification of outcome and selection bias [21]. The ROBINS-I tool is designed to assess the strengths and weaknesses of non-randomised studies on the effects of interventions in terms of their risk of bias. The tool views each study as an attempt to mimic a hypothetical pragmatic randomised trial, and covers seven distinct domains through which bias might be introduced: confounding, selection or participants into the study, classification of interventions, deviations due to intended interventions, missing data, measurement of outcome measures and selection of the reported result. The systematic review was registered on the EU Register of Post-Authorisation Studies (EUPAS18909) and reported according to PRISMA (Preferred Reporting Items for Systematic Reviews) [22].

### Data extraction

Part of the difficulty in interpreting the results from existing studies can relate to confounding by indication whereby the indication for treatment is itself associated with the risk of future health outcomes. Attempts to circumvent the problems of confounding by indication potentially include using different comparator or reference groups for analysis (for example, using alternative antidepressants as the comparator) and different study designs. For this reason, data from included studies were extracted for the following characteristics: study design, sample size, type of comparator or reference group reported, and the accuracy and completeness of information on confounders (including the severity of depression, indication for treatment, lifestyle factors, use of co-prescribed medication, maternal age at conception and family history). For each comparison, crude and adjusted effect estimates (odds ratios, hazard ratios and rate ratios) were identified with corresponding 95% confidence intervals (CIs). For some studies, only the adjusted effect estimates were available. The outcomes of interest were the risk of ASD and ADHD in children following antidepressant exposure during pregnancy.

#### Comparators, reference groups and sibling study design

To examine the impact of using different comparator or reference groups in causal inference, effect estimates for the following pre-specified antidepressant comparator or reference groups were extracted: (1) maternal exposure during pregnancy vs. all unexposed women (we refer to this as the classical comparison), (2) maternal exposure during the pre-pregnancy period vs. all unexposed women (this exposure should theoretically be non-causal and may act as a negative control), (3) maternal exposure during pregnancy vs. all unexposed women restricted to those with a history of affective disorder (such as depression, anxiety and bipolar disorder) (the restriction may remove some confounding by indication or severity in that the indication for treatment is independently associated with ASD/ADHD, for example, through genetics), (4) paternal exposure during the maternal pregnancy period vs. all unexposed women (this exposure should theoretically be non-causal and may act as a negative control) and (5) effect estimates from within-family sibling analyses (which accounts for all time-fixed within-family confounding). Exposure windows were as defined by the eligible studies.

### Analysis

The characteristics of the studies included and heterogeneity in confounding adjustment were first described. Effect estimates from each study were then used to reproduce the results from the meta-analysis by Man et al. and to assess the effect of adding effect estimates from additional published studies that reported associations using a classical reference group consisting of all unexposed women [15]. Crude and adjusted effect estimates were calculated on the natural log scale and pooled using the generic inverse variance method of analysis. Random-effects models were generated with fixed-effect models as the sensitivity analysis. This approach was then used to explore the pre-specified comparisons or reference groups that were compared to those from the classical reference group. Studies published using the same national data sources and patients were included separately and effect estimates generated with each separate study included as a sensitivity analysis. A leave-one-out analysis was also conducted when three or more studies were pooled to test the robustness of the analysis.

Odds ratios from case–control studies and hazard ratios from cohort studies were combined because they closely approximate each other, with sensitivity analyses performed by study design to assess for heterogeneity [23, 24]. Effect estimates were pooled by trimester of exposure where this information was available. When estimates for SSRI exposure and any antidepressant exposure were each reported within the same study, the effect estimate for SSRI exposure was preferentially used because it is the most widely used group of antidepressants, with sensitivity analysis performed using estimates for any antidepressant exposure. For reporting, pooled effect estimates are subsequently referred to as risk ratios throughout. Analyses were conducted in Review Manager 5.3 (Copenhagen: The Nordic Cochrane Centre, The Cochrane Collaboration, 2014). Publication bias was assessed by testing for funnel-plot asymmetry using the Egger test for studies reporting results of the classical reference group with ASD and ADHD.

## Results

This systematic review identified 464 articles (111 articles from MEDLINE and 353 articles from EMBASE).

### Autism spectrum disorder

Following the removal of duplicates and screening of remaining articles, there was a total of 15 observational studies measuring ASD as an outcome [16–19, 25–35]. No additional studies were identified from the cumulative review requested by the PRAC and submitted by the marketing authorisation holders of SSRIs. Characteristics of the included studies are presented in Table 1. For ASD as the outcome, seven case–control studies (involving 291,468 patients and 13,243 cases of ASD) [17, 18, 26–29, 32] and eight cohort studies (involving 3,294,218 patients and 27,342 cases of ASD) [16, 19, 25, 30, 31, 33–35] were included.

**Table 1:** Characteristics of included observational studies measuring risk of ASD and ADHD with antidepressant exposure in pregnancy

Study [Reference]	Primary study design	Number of children	Number of cases	Case definition	Data source, country (period)
Autism spectrum disorder					
Boukhris 2015 [16]	Cohort	145,456	1054	ICD-9, ICD-10	Quebec pregnancy children’s cohort, Canada (1998–2009)
Brown 2017 [23]	Cohort	35,906	394	ICD-9, ICD-10	Administrative databases, Ontario, Canada (2002–2010)
Castro 2016 [17]	Case–control	4650	1245	ICD-9	Massachusetts General Hospital, USA (1997–2010)
Clements 2015 [18]	Case–control	5399	1377	ICD-9	Massachusetts General Hospital, USA^a^ (1997–2010)
Croen 2011 [24]	Case–control	1805	298	ICD-9	Kaiser Permanente, USA (1995–1999)
El Marroun 2014 [19]	Cohort	5976	Not reported	*CBCL 1.5–5, SRS	Generation R study, Netherlands (2002–2006)
Eriksson 2012 [25]	Case–control	173,577	187	Clinical diagnosis	Stockholm, Sweden (2002–2006)
Gidaya 2014 [26]	Case–control	57,365	5215	ICD-10	Danish Civil Registration System, Denmark (1996–2006)
Harrington 2014 [27]	Case–control	966	492	ADI-R/ADOS	Charge study, USA (2003–2010)
Hviid 2013 [28]	Cohort	626,875	3892	ICD-10	Danish Civil Registration System, Denmark (1997–2005)
Malm 2016 [29]	Cohort	64,754	307	ICD-10	National registers, Finland (1996–2010)
Rai 2013 [30]	Case–control	47,706	4429	ICD-9, ICD-10	Regional administrative registries, Sweden (2001–2007)
Sorensen 2013 [31]	Cohort	655,615	5437	ICD-8, ICD-10	Danish Civil Registration System, Denmark (1996–2006)
Sujan 2017 [32]	Cohort	1,580,629	14,617	ICD-9, ICD-10	National administrative registries, Sweden (1996–2012)
Viktorin 2017 [33]	Cohort	179,007	1641	ICD-10	National administrative registries, Sweden (2006–2007)
Attention deficit hyperactivity disorder					
Castro 2016 [17]	Case–control	5498	1701	ICD-9	Massachusetts General Hospital, USA (1997–2010)
Clements 2015 [18]	Case–control	7874	2243	ICD-9	Massachusetts General Hospital, USA^a^ (1997–2010)
Figueroa 2010 [34]	Case–control	38,572	431	ICD-9	Marketscan, USA (1997–2006)
Laugesen 2013 [35]	Cohort	877,778	12,841	ICD-8, ICD-10	Danish Civil Registration System, Denmark (1996–2009)
Malm 2016 [29]	Cohort	64,754	514	ICD-10	National registers, Finland (1996–2010)
Man 2017 [36]	Cohort	190,618	5659	ICD-9	Clinical Data Analysis & Reporting System, Hong Kong (2001–2009)
Sujan 2017 [32]	Cohort	1,580,629	32,924	ICD-9, ICD-10	National administrative registries, Sweden (1996–2012)

*Parent-reported autistic symptoms assessed using: CBCL 1.5–5, the pervasive developmental problems subscale of the Child Behaviour Checklist for ages 1.5–5, and SRS, the Social Responsiveness Scale

“Clinical diagnosis” indicates receiving intervention for ASD at a specialised Autism Centre for Young Children

ADI-R Autism Diagnostic Interview, Revised, ADOS Autism Diagnostic Observation Schedule, ICD International Classification of Disease coding system

^a^New, independent cohort

Of the 15 observational studies measuring ASD as the outcome, all reported adjusted effect estimates for the comparison between maternal antidepressant exposure during pregnancy vs. all unexposed women (nine of which reported effect estimates by trimester). Eight observational studies reported adjusted effect estimates for maternal antidepressant exposure during the pre-pregnancy period vs. all unexposed women. Seven observational studies reported adjusted effect estimates for maternal antidepressant exposure during pregnancy vs. all unexposed women with a past medical history of affective disorder. Three observational studies reported results from a sibling analysis [25, 33, 35] and two observational studies reported results for paternal antidepressant exposure during the maternal pregnancy period vs. all unexposed women [33, 34] (Additional file 1: Table S1).

### Attention deficit hyperactivity disorder

**Fig. 1:**
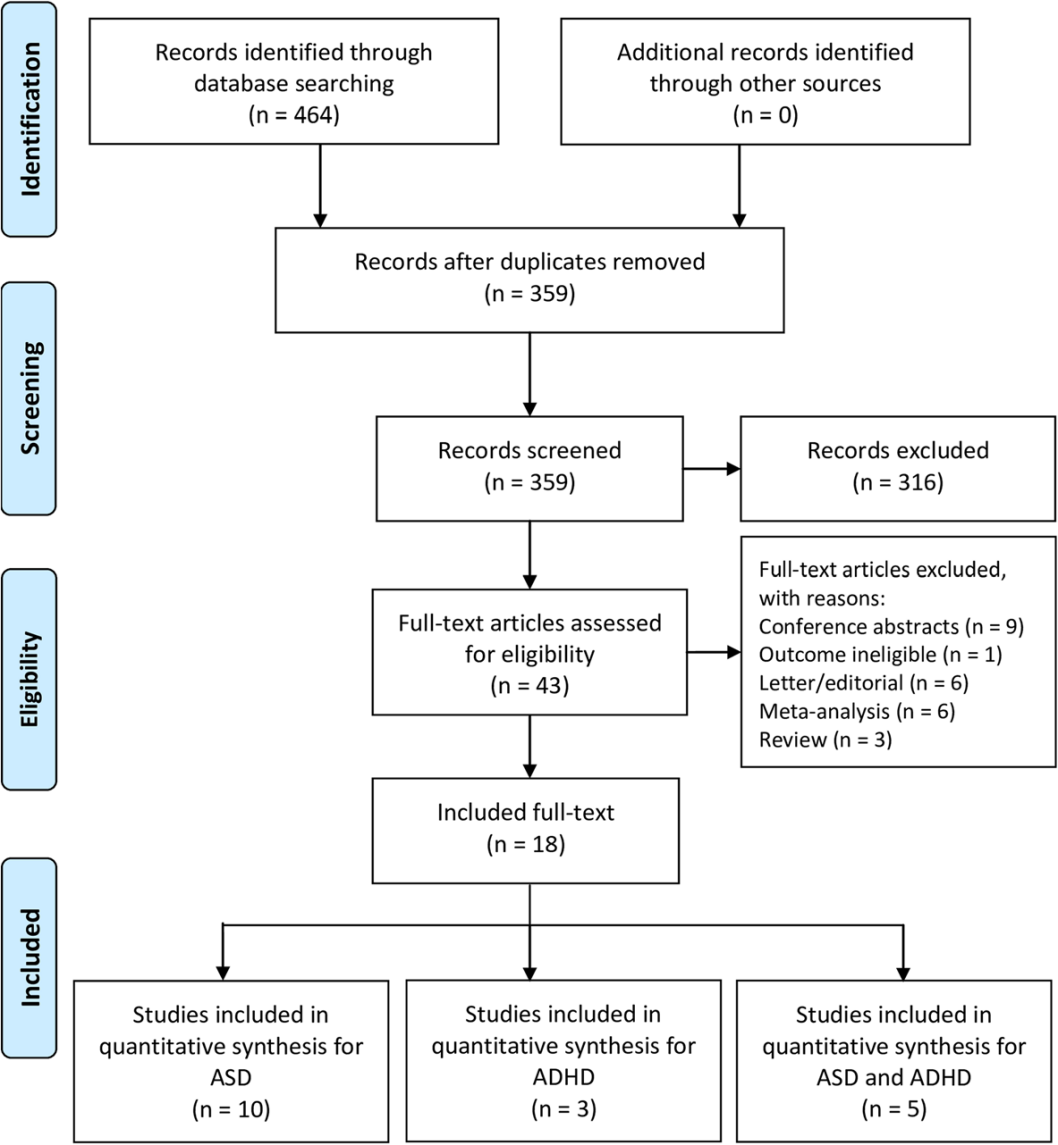
PRISMA (Preferred Reporting Items for Systematic Reviews) flow diagram for study selection. ADHD attention deficit hyperactivity disorder, ASD autism spectrum disorder

Following the removal of duplicates and screening of remaining articles, a total of seven observational studies measuring ADHD as an outcome [17, 18, 31, 34, 36–38] were identified (Fig. 1). For ADHD as the outcome, three case–control studies (involving 51,944 patients and 4,375 cases of ADHD) [17, 18, 36] and four cohort studies (involving 2,713,779 patients and 51,938 cases of ADHD) [31, 33, 37, 38] were included. Of the seven included observational studies measuring ADHD as the outcome, all reported adjusted effect estimates for the association between maternal antidepressant exposure during pregnancy vs. all unexposed women (of which six reported effect estimates by trimester). Five observational studies reported adjusted effect estimates for maternal antidepressant exposure during the pre-pregnancy period vs. all unexposed women. One observational study reported effect estimates for maternal antidepressant exposure during pregnancy vs. unexposed women with a past medical history of affective disorder [31]. Three observational studies reported results from sibling analyses [34, 37, 38] and one study reported estimates for paternal antidepressant exposure during pregnancy [34] (Additional file 1: Table S1).

#### Confounding factors assessed by the included observational studies

For ASD as an outcome, all studies evaluated the impact of maternal age and maternal psychiatric history (Table 2). Most studies evaluated sex of the offspring, year of birth, gestational age, birth weight, educational attainment, ethnicity or country of origin, and parity. Adjustment for use of other medicines during pregnancy occurred in five studies assessing ASD as the outcome and four studies assessing ADHD as the outcome [19, 25, 31, 34–37]. However, the definition of exposure to the use of other medicines during pregnancy focused on the use of other psychotropic drugs [35, 36, 38], use of anxiolytics and sedatives [19, 31, 37] or use of nicotine products [29], with only one study accounting for all types of medications used [25]. Adjustment for paternal psychiatric history, maternal physical history, complications in pregnancy, smoking status, and alcohol or other substance misuse occurred less often. The severity of depression was evaluated in only four studies and was limited either to measures of maternal treatment intensity in terms of number of antidepressant and psychotherapy visits [17, 18] or to use of a validated self-reported questionnaire [19], and by applying high-dimensional propensity scores [25]. Similar distributions were seen for ADHD as an outcome (Table 3). None of the included studies adjusted for the recurrent nature or the duration of depression, and no studies used diagnoses recorded in primary care, potentially leading to under-ascertainment of depression history.

**Table 2:** Confounders and risk factors evaluated in studies of maternal antidepressant use during pregnancy and risk of autism spectrum disorder in offspring

Potential confounder	Boukhis 2015	Brown 2017^d^	Castro 2016	Clements 2015	Croen 2011	El Marroun 2014	Eriksson 2012	Gidaya 2014	Harrington 2014	Hviid 2013	Malm 2016	Rai 2013	Sorensen 2013	Sujan 2017	Viktorin 2017
Reference	16	23	17	18	24	19	25	26	27	28	29	30	31	32	33
Gender	Y	Y	Y	Y	Y	Y	Y	Y	Y	Y	Y	Y	Y	–	–
Year of birth	Y	–	Y	Y	Y	–	–	Y	Y	Y	Y	Y	–	Y	Y
Birth weight	–	–	–	Y	Y	–	–	Y	–	–	Y	Y	Y	–	–
Gestational age at delivery	–	Y	Y	Y	Y	Y	–	Y	–	Y	Y	Y	Y	–	–
Maternal age	Y	Y	Y	Y	Y	Y	Y	Y	Y	Y	Y	Y	Y	Y	Y
Marital status	Y	–	–	–	–	–	–	–	–	–	Y	–	–	–	–
Living alone	Y	–	–	–	–	–	–	–	–	–	–	–	–	–	–
Education	Y	–	Y	Y	Y	Y	–	Y	Y	Y	–	Y	–	Y	–
Social assistance or care	Y	–	–	–	–	–	–	–	–	–	–	–	–	–	–
Maternal psychiatric history^a^	Y	Y	Y	Y	Y	Y	Y	Y	Y	Y	Y	Y	Y	Y	Y
Paternal psychiatric history^a^	Y	–	–	–	–	–	–	–	–	–	–	Y	Y	–	Y
Maternal physical history^b^	Y	Y	–	Y	–	–	–	Y	–	–	Y	–	–	–	–
Pre-pregnancy related/delivery	–	Y	–	Y	–	–	Y	Y	–	–	Y	–	–	–	–
Paternal physical history	Y	–	–	–	–	–	–	Y	–	–	–	–	–	–	–
Severity of depression	–	Y	Y	Y	–	Y	–	–	–	–	–	–	–	–	–
Smoking status	–	–	–	–	–	Y	–	–	–	Y	Y	Y	–	–	–
Alcohol or substance misuse	–	–	–	–	–	Y	–	–	Y	–	Y	Y	–	–	Y
Parity	–	Y	Y	Y	Y	–	–	Y	–	Y	Y	Y	Y	Y	–
Insurance type	–	–	Y	Y	–	–	–	–	–	–	–	–	–	–	–
Ethnicity or country of origin	–	–	Y	Y	Y	Y	–	–	Y	Y	Y	Y	–	Y	–
Maternal income	–	Y	Y	–	–	–	–	Y	–	–	–	–	–	–	–
Residence	–	–	–	–	–	–	–	–	–	Y	Y	–	–	–	–
Employment status	–	–	–	–	–	–	–	–	–	Y	Y	Y	–	–	–
Depression recurrence	–	–	–	–	–	–	–	–	–	–	–	–	–	–	–
Drugs other than antidepressants^c^	–	Y	–	–	–	Y	–	–	Y	–	Y	–	–	–	Y

^a^Heterogeneity in definitions used

^b^Includes only a limited number of physical conditions, such as diabetes, hypertension and autoimmune diseases, and varies by study

^c^Includes only a limited number of other drugs used during pregnancy and varies by study (benzodiazepines, antipsychotics, teratogens etc.)

^d^Combined covariates through high-dimensional propensity scores

**Table 3:** Potential confounders and risk factors evaluated in studies of maternal antidepressant use during pregnancy and risk of attention deficit hyperactivity disorder in offspring

Potential confounder	Castro 2016	Clements 2015	Figueroa 2010	Laugesen 2013	Malm 2016	Man 2017	Sujan 2017
Reference	17	18	34	35	29	36	32
Gender	Y	Y	Y	Y	Y	Y	–
Year of birth	Y	Y	Y	–	Y	Y	Y
Birth weight	–	Y	Y	Y	Y	Y	–
Gestational age at delivery	Y	Y	Y	Y	Y	Y	–
Maternal age	Y	Y	Y	Y	Y	Y	Y
Marital status	–	–	–	Y	Y	–	–
Living alone	–	–	–	–	–	–	–
Education	Y	Y	–	–	–	–	Y
Social assistance or economic status	–	–	–	–	–	Y	–
Maternal psychiatric history^a^	Y	Y	Y	Y	Y	Y	Y
Paternal psychiatric history^a^	–	–	–	Y	–	–	–
Maternal physical history^b^	–	Y	Y	Y	Y	Y	–
Pregnancy or delivery complications	–	Y	Y	–	Y	–	–
Paternal physical history	–	–	Y	–	–	–	–
Severity of depression	Y	Y	–	–	–	–	–
Smoking status	–	–	–	Y	Y	–	–
Alcohol or substance misuse	–	–	–	–	Y	–	–
Parity	Y	Y	–	Y	Y	Y	Y
Insurance type	Y	Y	–	–	–	–	–
Ethnicity or country of origin	Y	Y	–	–	Y	–	Y
Maternal income	Y	–	–	–	–	–	–
Residence	–	–	Y	–	Y	–	–
Employment status	–	–	–	–	Y	–	–
Depression recurrence	–	–	–	–	–	–	–
Drugs other than antidepressants^c^	–	–	Y	Y	Y	Y	–

^a^Heterogeneity in definitions used

^b^Includes only a limited number of physical conditions, such as diabetes, hypertension and autoimmune disease, and varies by study

^c^Includes only a limited number of other drugs used during pregnancy and varies by study (benzodiazepines, antipsychotics, teratogens etc.)

#### Impact of meta-analysing effect estimates

##### Autism spectrum disorder

**Fig. 2:**
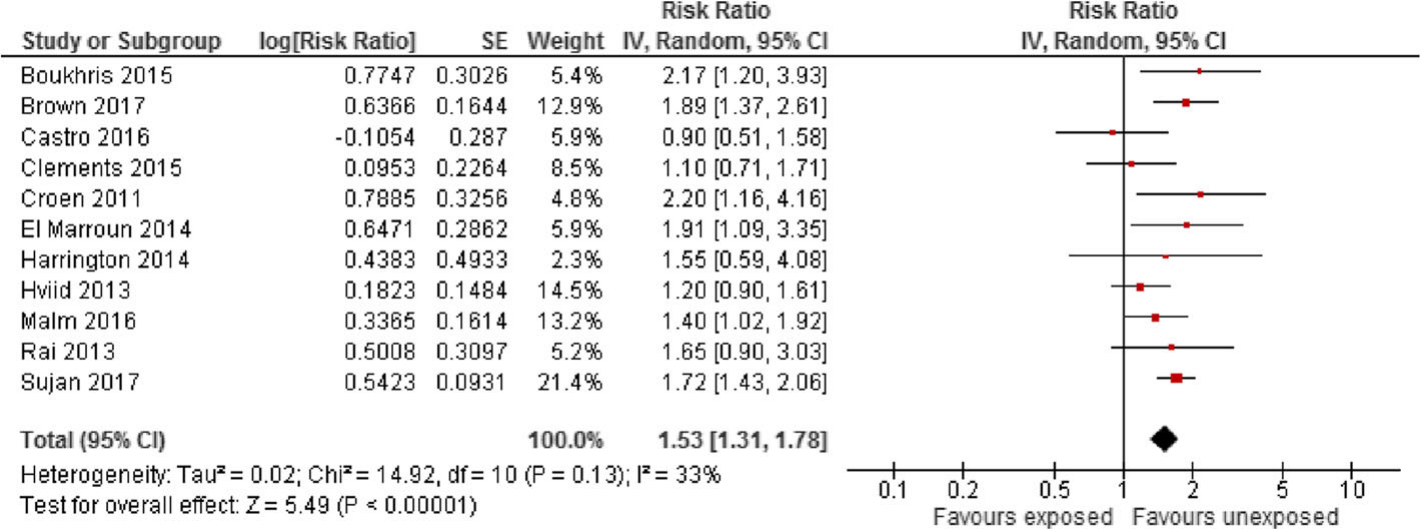
Risk of autism spectrum disorder associated with maternal antidepressant exposure during pregnancy compared to unexposed women. CI confidence interval, df degrees of freedom, SE standard error of the mean

The meta-analysis by Man et al. demonstrating that maternal SSRI exposure during pregnancy was associated with a significantly increased risk of ASD in children vs. all unexposed women was successfully replicated (Additional file 2: Figure S1) [15]. Effect estimates were then calculated incorporating data from the classical reference group consisting of all unexposed women using additional published articles on antidepressant use during pregnancy. Following inclusion of these data, the risk of ASD with maternal antidepressant exposure during pregnancy vs. all unexposed women still appeared statistically significantly increased (random-effects adjusted risk ratio [RaRR] 1.53, 95% CI 1.31–1.78, Table 4 and Fig. 2). When evaluated by trimester of pregnancy, the risk of ASD in children with maternal antidepressant exposure during pregnancy vs. all unexposed women was statistically significantly elevated in the first and second trimesters only (Additional file 3: Figure S2).

**Table 4:** Pooled crude and adjusted effect estimates for the different comparator and reference groups in included observational studies measuring the association between antidepressant exposure and risk of autism spectrum disorder (random-effects model)

Comparison	Crude RR	No. of studies	Adjusted risk ratio	No. of studies
Maternal exposure during pregnancy vs. unexposed women	1.85 (1.60–2.23)	10	1.53 (1.31–1.78)	10
Maternal exposure pre-pregnancy vs. unexposed women	1.71 (1.42–2.05)	6	1.48 (1.29–1.71)	7
Maternal exposure during pregnancy vs. unexposed women with a history of affective disorder	1.35 (0.75–2.44)	3	1.18 (0.91–1.52)	6
Sibling study design	1.01 (0.48–2.14)	2	0.96 (0.65–1.42)	3
Paternal exposure during the maternal pregnancy period vs. unexposed women	1.40 (1.10–1.80)	1	1.29 (1.08–1.53)	2

Not all studies reported crude effect estimates explaining the difference in the number of studies. Pooled effect estimates are presented when reported by two or more studies

**Fig. 3:**
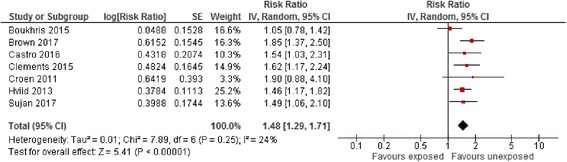
Risk of autism spectrum disorder associated with pre-pregnancy maternal antidepressant exposure during pregnancy compared to unexposed women. CI confidence interval, df degrees of freedom, SE standard error of the mean

**Fig. 4:**
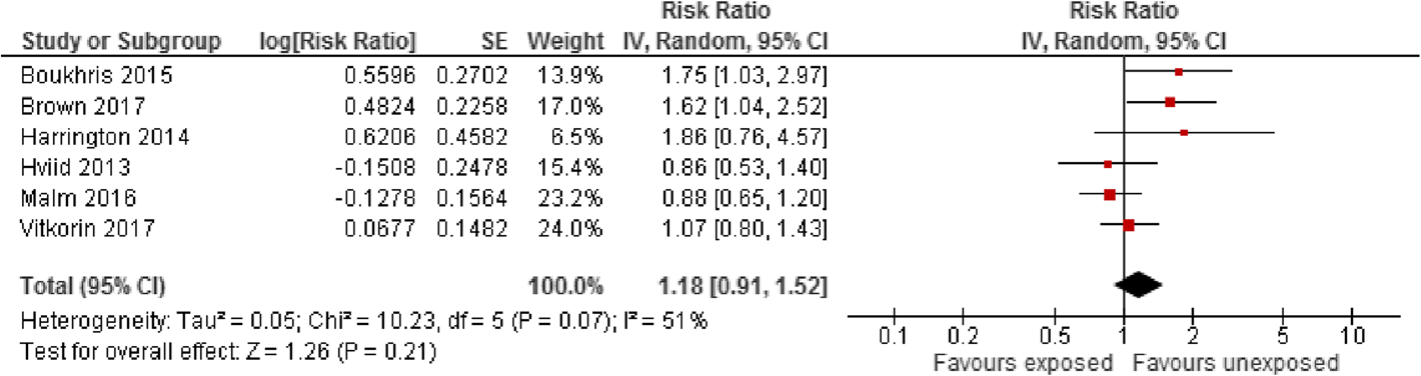
Risk of autism spectrum disorder associated with maternal antidepressant exposure during pregnancy compared to unexposed women with a history of affective disorder. CI confidence interval, df degrees of freedom, SE standard error of the mean

Pooled effect estimates for the risk of ASD among different comparator groups are summarised in Table 4. The risk of ASD associated with maternal antidepressant exposure during the pre-pregnancy period vs. all unexposed women appeared statistically significantly elevated (RaRR 1.48, 95% CI 1.29–1.71, Fig. 3) and was similar in size to that of exposure during pregnancy. Pooled effect estimates for the risk of ASD associated with maternal antidepressant exposure during pregnancy compared to unexposed women with a history of affective disorder did not appear to be statistically significantly increased (RaRR 1.18, 95% CI 0.91–1.52, Fig. 4). The effect estimate for the risk of ASD associated with maternal antidepressant exposure during pregnancy reported by three within-family sibling studies did not suggest an increase in risk (RaRR 0.96, 95% CI 0.65–1.42). In contrast, the effect estimate for the risk of ASD associated with paternal antidepressant exposure during the maternal pregnancy period vs. all unexposed women two studies showed a statistically significantly increased risk (RaRR 1.29, 95% CI 1.08–1.53).

##### Attention deficit hyperactivity disorder

**Fig. 5:**
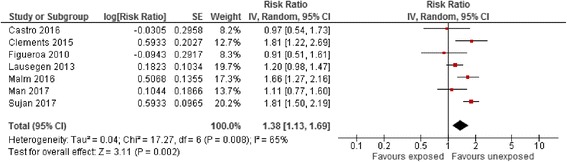
Risk of attention deficit hyperactivity disorder associated with maternal antidepressant exposure during pregnancy compared to unexposed women. CI confidence interval, df degrees of freedom, SE standard error of the mean

**Fig. 6:**
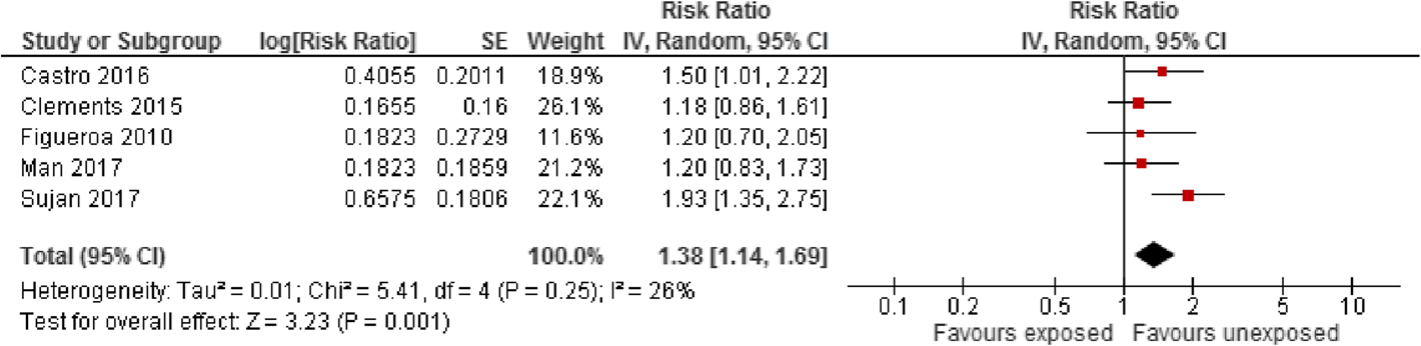
Risk of attention deficit hyperactivity disorder associated with pre-pregnancy maternal antidepressant exposure during pregnancy compared to unexposed women. CI confidence interval, df degrees of freedom, SE standard error of the mean

Effect estimates for the risk of ADHD among the different comparator groups are summarised in Table 5. The risk of ADHD with maternal antidepressant exposure during pregnancy using a classical reference group consisting of all unexposed women appeared to be statistically significantly increased (RaRR 1.38, 95% CI 1.13–1.69, Fig. 5). When evaluated by trimester of pregnancy, the risk of ADHD in children with maternal antidepressant exposure during pregnancy vs. all unexposed women was statistically significantly elevated in the first and second trimesters only using a random effect model (Additional file 4: Figure S3). The risk of ADHD associated with maternal antidepressant exposure during the pre-pregnancy period vs. all unexposed women was similarly statistically significantly elevated (RaRR 1.38, 95% CI 1.14–1.69, Fig. 6) and was similar in size to exposure during pregnancy.

**Table 5:** Pooled crude and adjusted effect estimates for the different comparator and reference groups in included observational studies measuring the association between antidepressant exposure and risk of attention deficit hyperactivity disorder (random-effects)

Comparison	Crude risk ratio	No. of studies	Adjusted risk ratio	No. of studies
Maternal exposure during pregnancy vs. unexposed women	2.04 (1.62–2.56)	5	1.38 (1.13–1.69)	7
Maternal exposure pre-pregnancy vs. unexposed women	1.42 (1.09–1.84)	2	1.38 (1.14–1.69)	5
Maternal exposure during pregnancy vs. unexposed women with a history of affective disorder	1.01 (0.80–1.27)	1	0.98 (0.77–1.24)	1
Sibling design	0.8 (0.5 to 1.2)	1	0.88 (0.70–1.11)	3
Paternal exposure during the maternal pregnancy period vs. unexposed women	–	0	1.71 (1.31–2.23)	1

Not all studies reported crude effect estimates explaining the difference in the number of studies. Pooled effect estimates are presented when reported by two or more studies

**Fig. 7:**
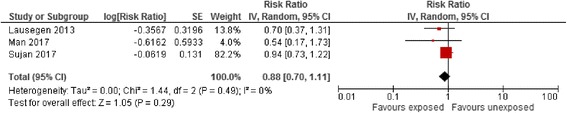
Risk of attention deficit hyperactivity disorder associated with maternal antidepressant exposure during pregnancy using a sibling study design. CI confidence interval, df degrees of freedom, SE standard error of the mean

The risk of ADHD associated with maternal antidepressant exposure during pregnancy vs. all unexposed women with a history of affective disorder from only a single cohort study suggested no significant association (RaRR 0.98, 95% CI 0.77–1.24). The effect estimate for the risk of ADHD associated with maternal antidepressant exposure during pregnancy reported in three within-family sibling studies suggested no significant association (RaRR 0.88, 95% CI 0.70–1.11, Fig. 7). A single observational study measuring the risk of ADHD associated with paternal antidepressant exposure during the maternal pregnancy period vs. all unexposed women was also statistically significant (RaRR 1.71, 95% CI 1.31–2.23).

##### Sensitivity analyses

The observed pattern of effect estimates for the other comparison and reference groups and sibling designs were similar when analysed using a fixed-effect model, although the association was significant in all trimesters for ASD and ADHD (Additional file 1: Table S2 and S3, Additional file 7: Fig. S4 and Additional file 8: Fig. S5).. Three studies used the same data source and related patients from Denmark [28, 30, 33] and three from Sweden [32, 34, 35]. The relative size of effect estimates between the different comparator groups was similar when studies with overlapping data from each country were substituted (Additional file 1: Table S4). Substituting the effect estimates for any antidepressant exposure from studies reporting estimates for both SSRI exposure and any antidepressant exposure produced very similar results (Additional file 1: Table S5). The risk of ASD and ADHD with maternal antidepressant exposure during pregnancy vs. all unexposed women and the observed pattern of effect estimates between different comparison and reference groups appeared similar across study designs (Additional file 1: Tables S6 and S7, Additional file 5: Figure S6 and Additional file 6: Figure S7). Results from a leave-one-out analysis were consistent with the main findings.

#### Risk of bias

Of the 15 studies assessing ASD as an outcome, 11 studies were considered to be at moderate risk of bias for the classical comparison between exposed and unexposed women and four studies at serious risk of bias (Additional file 1: Table S8). Of the five studies assessing ADHD as an outcome, all were considered to be at moderate risk of bias for the classical comparison between exposed and unexposed women (Additional file 1: Table S9). Specific areas judged to be affected related to: bias due to deviations from intended interventions (bias that arises when there are systematic differences between experimental intervention and comparator groups in the care provided, which represents a deviation from the intended intervention), bias due to baseline confounding and bias due to missing data. Pregnancy exposure windows were well defined in all studies although it was not possible to determine what proportion of patients with pre-pregnancy exposure continued antidepressant exposure during pregnancy. No evidence of publication bias was detected when testing for funnel-plot asymmetry using the Egger test for either ASD (*p* value = 0.433) or ADHD (*p* value = 0.901).

## Discussion

This systematic review was performed to evaluate the content of published observational studies investigating the association between antidepressant exposure during pregnancy and risk of ASD and ADHD in offspring. These studies can be difficult to interpret due to limitations in unmeasured confounding, particularly in relation to confounding by indication and the severity of depression, inherent in this type of research. We also evaluated which effect estimates associated with different comparator or reference groups have been reported and their potential for helping to interpret the results of such studies including pre-pregnancy exposure, restriction to women with a history of psychiatric illness, paternal exposure and sibling designs. Taken together, these results suggest that the significant associations observed using a classical reference group comparing risk among all unexposed women result from residual unmeasured confounding.

Variation in the type of confounding adjustment undertaken between observational studies existed, which may lead to heterogeneity and the risk of residual confounding. This partly relates to the limitations of currently available data sources for studying such effects and environmental and genetic influences. These are important factors when considering the limitations of undertaking potential meta-analyses with these data, as simply adding more results from similarly designed studies is unlikely to remove the problems of confounding and bias, but risks misleading by giving the appearance of better precision.

We reproduced data from an early meta-analysis that reported a significant association between maternal SSRI exposure during pregnancy and risk of ASD in offspring, observed when a classical reference group was used (defined purely by the absence of SSRI exposure in women) [15]. When this meta-analysis was updated with further published data, antidepressant exposure during pregnancy still appeared to be associated with a significantly increased risk of ASD in offspring when exposed women were compared to all unexposed women. However, the size of the association was more attenuated, falling from an association suggesting an 80% increased risk of ASD to an association suggesting a 53% increased risk. This supports our statement that simply adding more results from studies with similar limitations is unlikely to remove the problem of residual confounding. Women included in the classical reference group are more likely to be healthy, without a history of affective disorder and the social, environmental and genetic factors associated with these. Such comparisons are at high risk of confounding by indication and severity of illness, and it is difficult to determine with confidence whether associations from this type of comparison alone are causal because many factors remain unmeasured. Similarly, we observed an association suggesting a 38% increased risk of ADHD with antidepressant exposure during pregnancy.

We then examined which effect estimates had been reported using different reference groups where exposure may or may not theoretically cause ASD or ADHD, and then examined the effect of pooling these estimates between studies. The most commonly reported alternative comparison was for pre-pregnancy exposure. For both ASD and ADHD as an outcome, the size of effect estimates for antidepressant exposure during pre-pregnancy were comparable to that observed for exposure during pregnancy using the classical reference group consisting of all unexposed women. Additionally, paternal exposure during the maternal pregnancy period was also associated with an increased risk of ASD and ADHD in offspring, although this was less frequently assessed. The next commonly reported comparison involved restricting the comparator group to women with a history of affective disorder, in an attempt to circumvent unmeasured confounding and confounding by indication by design. These effect estimates appeared to be non-significant for both ASD and ADHD. Finally, sibling studies are a type of within person (mother) design that can circumvent fixed confounding and some confounding by indication, which may affect between-person comparisons. These sibling studies found no statistically significant association with ASD or ADHD.

A systematic review by Brown et al. was recently published focusing specifically on the risk of ASD from SSRI exposure in pregnancy and provides complimentary information [39]. Brown et al. examined the impact of studies adjusting for current and past psychiatric history, and the impact of restricting the population to women with a history of psychiatric illness. They combined these data using fixed and random-effects models. The quality of the included observational studies was assessed using the SAQOR quality rating scale, a tool that also attempts to capture confounding by indication [40]. Assessing quality using this separate approach, Brown et al. similarly noted that none of the included case–control studies met the criteria for adequate consideration of distorting influences. We used the recently developed ROBINs-I tool to assess the risk of bias of the included observational studies, and noted similar issues. To our knowledge there are no head-to-head comparisons between these different quality assessment tools to determine which is best. However, consistency in results using different tools may help to provide independent validation of the conclusions of both studies on this topic.

Brown et al. similarly noted the increased association between SSRI exposure in pregnancy compared to all unexposed women, even following an adjustment for a history of psychiatric illness, which suggests that current approaches that simply rely upon confounding adjustment between these populations is insufficient. However, only two studies examining the impact of restriction of the population were available, both of which were non-significant. The potential for under-identification of maternal illness was also noted, because diagnoses were defined using administrative databases only. As in our analysis, in many instances these diagnoses are hospital based and probably more at the severe end of the spectrum, which may partly explain the observed pattern of results seen in our study.

### Limitations of the studies reviewed and the methodological approach

While there is a temptation to obtain a single result from combining the results of studies, the methods used for combining them treat them in the same way as if the data had come from randomised trials. The uncertainty in estimates derived from observational studies is potentially greater than the sampling error that is captured by a confidence interval and may be related to heterogeneity, such as variation in confounding adjustment undertaken between studies. It is perhaps a matter of debate as to whether such studies should routinely be meta-analysed and if so which type of model or comparisons should be used. For example, although the I^2^ statistic is often reported in meta-analyses as a way of detecting heterogeneity, it is not an absolute measure of heterogeneity and does not communicate the predicted range of effects [41].

Caution is generally required when combining estimates from case–control and cohort studies in a meta-analysis because this may be an important source of heterogeneity [42]. The dominant issue influencing both study designs is unmeasured confounding, largely weighted by confounding indication and by severity of disease. This affects case–control and cohort studies similarly but the technique allows more studies to be combined to highlight the observed effect, in keeping with other similar results comparing these study designs [43]. A strength of our study is the inclusion of a large number of studies incorporating other types of comparators and designs, comparing the effects in both ASD and ADHD. However, for some alternative comparison groups, only a limited number of effect estimates were identified, such as paternal antidepressant exposure during the maternal pregnancy period, and their potential utility requires further evaluation, although the sibling design has good potential.

General limitations with most of the observational studies included in this systematic review include incomplete ascertainment of psychiatric history (i.e. diagnoses only managed in primary care) and inadequate adjustment for the severity of psychiatric depression, which may be related to the outcomes of interest. Outcomes were also predominantly defined using electronic coding within population health-care databases, which still largely require to be validated. For example, ASD represents a spectrum of symptoms and diagnoses that may not be fully captured using standard coding approaches and may require the use of validated scales to assess a more complete range of neurodevelopmental disorders. This potentially increases the chance of outcome misclassification and either underestimating or overestimating risk. Accurate exposure assessment is also required for such studies and it was not reported what proportion of patients with pre-pregnancy exposure may have continued antidepressant exposure during pregnancy. One observational study clearly reported in the definition of pre-pregnancy exposure that the participants were subsequently unexposed during pregnancy and the reported effect estimate for the association between pre-pregnancy exposure and risk of ASD was still statistically significantly elevated. Similarly, a correlation may exist between paternal and maternal antidepressant exposure during pregnancy.

Sample size limitations mean it is currently challenging to conduct observational studies assessing the association between certain neurodevelopmental disorders and certain antidepressants, particularly for individual medicines where there are differences in the frequency of use between countries [14]. Such analyses may require the use of multiple population databases to improve power and precision if existing limitations in study design and confounding adjustment can be overcome. Most analyses failed to account for exposure to other commonly used medicines and of those which did, only limited types of exposure were evaluated. Further adjustment for such medication use may help to provide information related to severity of the underlying condition and overall health status. Our search is limited by the databases used, as we did not have access to the full range potentially available. Lastly, another potential limitation is that the authors of the studies included were not contacted.

## Conclusions

Existing observational studies measuring the association between antidepressant exposure during pregnancy and risk of ASD and ADHD in offspring are heterogeneous in their design and method of analysis. Classical comparisons between exposed and all unexposed women are at risk of residual confounding that is difficult to adjust for fully with existing data sources. Other comparisons and study designs, such as sibling studies, contribute important additional information. It is recommended that such future studies should consider incorporating these types of analysis routinely in the design. However, the limitations of undertaking meta-analyses using such data and the value of using other comparators, reference groups and other study designs requires further methodological exploration. This has important implications because such observational findings may result in significant public health concerns and media interest, with potential consequences of cessation of antidepressant treatment during pregnancy and its impact on both maternal and child health, and in this regard the benefit risk should be considered in terms of both mother and offspring.

## Additional files

Additional file 1: Table S1. Variation in comparison and reference groups analysed among observational studies measuring the association between antidepressant exposure during pregnancy and risk of ASD and ADHD in offspring. **Table S2.** Crude and adjusted effect estimates for the different comparator and reference groups in included observational studies measuring the association between antidepressant exposure and risk of ASD (fixed-effect model). **Table S3.** Pooled crude and adjusted effect estimates for the different comparator and reference groups in included observational studies measuring the association between antidepressant exposure and risk of ADHD (fixed-effect model). **Table S4.** Effect estimates when analyses were replaced with other studies using data from Denmark and Sweden. **Table S5.** Adjusted effect estimates for the different comparator groups in included studies measuring the association between antidepressant exposure and risk of ASD and ADHD substituting estimates for SSRI exposure with any antidepressant exposure. **Table S6.** Adjusted effect estimates for the different comparator groups in included studies measuring the association between antidepressant exposure and risk of ASD, by study design. **Table S7.** Adjusted effect estimates for the different comparator groups in included studies measuring the association between antidepressant exposure and risk of ADHD, by study design. **Table S8.** Risk of bias among primary studies measuring the association between maternal antidepressant exposure during pregnancy and risk of ASD in offspring. **Table S9.** Risk of bias among primary studies measuring the association between maternal antidepressant exposure during pregnancy and risk of ADHD in offspring. (DOCX 36 kb)

Additional file 2: Figure S1. Replication of study results from the previous meta-analysis by Man et al. [15]. (TIF 120 kb)

Additional file 3: Figure S2. Adjusted effect estimates for the risk of ASD associated with maternal antidepressant exposure during pregnancy compared to unexposed women by trimester. (TIF 304 kb)

Additional file 4: Figure S3. Adjusted effect estimates for the risk of ADHD associated with maternal antidepressant exposure during pregnancy compared to unexposed women by trimester. (TIF 285 kb)

Additional file 5: Figure S6. Adjusted effect estimates for the risk of ASD associated with maternal antidepressant exposure during pregnancy compared to unexposed women according to study design. (TIF 274 kb)

Additional file 6: Figure S7. Adjusted effect estimates for the risk of ADHD associated with maternal antidepressant exposure during pregnancy compared to unexposed women according to study design. (TIF 229 kb)

Additional file 7: Figure S4. Adjusted effect estimates for the risk of ASD associated with maternal antidepressant exposure during pregnancy compared to unexposed women by trimester (fixed-effect analysis). (TIF 301 kb)

Additional file 8: Figure S5. Adjusted effect estimates for the risk of ADHD associated with maternal antidepressant exposure during pregnancy compared to unexposed women by trimester (fixed-effect analysis). (TIF 281 kb)

## Abbreviations

ADHD: Attention deficit hyperactivity disorder
ADI-R: Autism Diagnostic Interview, Revised
ADOS: Autism Diagnostic Observation Schedule
ASD: Autism spectrum disorder
CI: Confidence interval
df: degrees of freedom
DSM-5: *Diagnostic and Statistical Manual of Mental Disorders 5*
EMA: European Medicines Agency
ICD: International Classification of Disease
PRAC: Pharmacovigilance Risk Assessment Committee
PRISMA: Preferred Reporting Items for Systematic Reviews
RaRR: Random-effects adjusted risk ratio
SE: Standard error of the mean
SSRI: Selective serotonin reuptake inhibitor
