# Supplementary material for: Antidepressant use during pregnancy and risk of autism spectrum disorder and attention deficit hyperactivity disorder: systematic review of observational studies and methodological considerations
Source: BMC Med. 2018 Jan 15;16:6. doi: 10.1186/s12916-017-0993-3 (PMC5767968; doi:10.1186/s12916-017-0993-3)
Supplement: Supplementary file 1 — Variation in comparison and reference groups analysed among observational studies measuring the association between antidepressant exposure during pregnancy and risk of ASD and ADHD in offspring. Table S2. Crude and adjusted effect estimates for the different comparator and reference groups in included observational studies measuring the association between antidepressant exposure and risk of ASD (fixed-effect model). Table S3. Pooled crude and adjusted effect estimates for the different comparator and reference groups in included observational studies measuring the association between antidepressant exposure and risk of ADHD (fixed-effect model). Table S4. Effect estimates when analyses were replaced with other studies using data from Denmark and Sweden. Table S5. Adjusted effect estimates for the different comparator groups in included studies measuring the association between antidepressant exposure and risk of ASD and ADHD substituting estimates for SSRI exposure with any antidepressant exposure. Table S6. Adjusted effect estimates for the different comparator groups in included studies measuring the association between antidepressant exposure and risk of ASD, by study design. Table S7. Adjusted effect estimates for the different comparator groups in included studies measuring the association between antidepressant exposure and risk of ADHD, by study design. Table S8. Risk of bias among primary studies measuring the association between maternal antidepressant exposure during pregnancy and risk of ASD in offspring. Table S9. Risk of bias among primary studies measuring the association between maternal antidepressant exposure during pregnancy and risk of ADHD in offspring. (DOCX 36 kb) [file 12916_2017_993_MOESM1_ESM.docx]

**Additional file 1**

**Table S1. Variation in comparison and reference groups analysed among observational studies measuring the association between antidepressant exposure during pregnancy and risk of ASD and ADHD in offspring.**

| Study  [Reference] | Maternal pregnancy exposure vs. all unexposed women | Maternal pregnancy exposure vs. all unexposed women by trimester | Maternal prepregnancy exposure vs. all unexposed women | Maternal pregnancy exposure vs. unexposed women with history of affective disorder | Sibling study  design | Paternal exposure during pregnancy period vs. all unexposed women |
| --- | --- | --- | --- | --- | --- | --- |
| **Autism spectrum disorder** | | | |  |  |  |
| Boukhris 2015 [16] | Yes | Yes | Yes | Yes | - | - |
| Brown 2017 [23] | Yes | Yes | Yes | Yes | Yes | - |
| Castro 2016 [17] | Yes | Yes | Yes | - | - | - |
| Clements 2015 [18] | Yes | Yes | Yes | - | - | - |
| Croen 2011 [24] | Yes | Yes | Yes | - | - | - |
| El Marroun 2014 [19] | Yes | - | - | - | - | - |
| Eriksson 2012 [25] | Yes | - | - | - | - | - |
| Gidaya 2014 [26] | Yes | Yes | Yes | - | - | - |
| Harrington 2014 [27] | Yes | Yes | - | Yes | - | - |
| Hviid 2013 [28] | Yes | Yes | Yes | Yes | - | - |
| Malm 2016 [29] | Yes | - | - | Yes | - | - |
| Rai 2013 [30] | Yes | - | - | - | - | - |
| Sorensen 2013 [31] | Yes | - | - | Yes | Yes | Yes |
| Sujan 2017 [32] | Yes | Yes | Yes | - | Yes | Yes |
| Viktorin 2017 [33] | Yes | - | - | Yes | - | - |
| **Attention Deficit Hyperreactivity Disorder** | | |  |  |  |  |
| Castro 2016 [17] | Yes | Yes | Yes | - | - | - |
| Clements 2015 [18] | Yes | Yes | Yes | - | - | - |
| Figueroa 2010 [34] | Yes | Yes | Yes | - | - | - |
| Laugesen 2013 [35] | Yes | Yes | - | - | Yes | - |
| Malm 2016 [29] | Yes | - | - | Yes | - | - |
| Man 2017 [36] | Yes | Yes | Yes | - | Yes | - |
| Sujan 2017 [32] | Yes | Yes | Yes | - | Yes | Yes |

**Table S2. Crude and adjusted effect estimates for the different comparator and reference groups in included observational studies measuring the association between antidepressant exposure and risk of ASD (fixed effect model).**

|  | Autism spectrum disorder | | | |
| --- | --- | --- | --- | --- |
| **Comparison** | **Crude RR** | **No.**  **studies** | **Adjusted RR** | **No.**  **studies** |
| Maternal exposure during pregnancy vs unexposed women | 1.94 (1.77-2.12) | 10 | 1.57 (1.41-1.75) | 10 |
| Maternal exposure pre-pregnancy vs unexposed women | 1.69 (1.50-1.89) | 6 | 1.48 (1.31-1.67) | 7 |
| Maternal exposure during pregnancy vs unexposed women with a history of affective disorder | 1.33 (1.07-1.64) | 3 | 1.12 (0.95-1.32) | 6 |
| Sibling study design | 1.00 (0.55-1.80) | 2 | 0.96 (0.65-1.42) | 3 |
| Paternal exposure during the maternal pregnancy period vs. unexposed women | 1.40 (1.10-1.80) | 1 | 1.29 (1.08-1.53) | 2 |

*Not all studies reported crude effect estimates explaining the difference in the number of studies. Pooled effect estimates are presented when reported by two or more studies.

**Table S3. Pooled crude and adjusted effect estimates for the different comparator and reference groups in included observational studies measuring the association between antidepressant exposure and risk of ADHD (fixed effect model).**

|  | Attention Deficit Hyperactivity Disorder | | | |
| --- | --- | --- | --- | --- |
| **Comparison** | **Crude RR** | **No.**  **studies** | **Adjusted RR** | **No.**  **studies** |
| Maternal exposure during pregnancy vs unexposed women | 2.15 (1.94-2.37) | 5 | 1.45 (1.31-1.62) | 7 |
| Maternal exposure pre-pregnancy vs unexposed women | 1.43 (1.17-1.75) | 2 | 1.38 (1.17-1.64) | 5 |
| Maternal exposure during pregnancy vs unexposed women with a history of affective disorder | 1.01 (0.80-1.27) | 1 | 0.98 (0.77-1.24) | 1 |
| Sibling design | 0.8 (0.5 to 1.2) | 1 | 0.88 (0.70-1.11) | 3 |
| Paternal exposure during the maternal pregnancy period vs. unexposed women | - | 0 | 1.71 (1.31-2.23) | 1 |

*Not all studies reported crude effect estimates explaining the difference in the number of studies. Pooled effect estimates are presented when reported by two or more studies.

**Table S4. Effect estimates when analyses were replaced with other studies using data from Denmark and Sweden.**

|  | Autism spectrum disorder | |
| --- | --- | --- |
| **Comparison** | **Adjusted RR** | **No. studies** |
| **Maternal exposure during pregnancy vs unexposed women** |  |  |
| Danish data sources |  |  |
| *With Gidaya 2014* |  |  |
| - Fixed-effect | 1.65 (1.49-1.84) | 10 |
| - Random-effects | 1.64 (1.44-1.86) | 10 |
| *With Sorensen 2013* |  |  |
| - Fixed-effect | 1.62 (1.46-1.80) | 10 |
| - Random-effects | 1.61 (1.43-1.81) | 10 |
| Swedish data sources |  |  |
| *With Rai 2013* |  |  |
| - Fixed-effect | 1.48 (1.30-1.70) | 10 |
| - Random-effects | 1.50 (1.27-1.77) | 10 |
| *With Viktorin 2017* |  |  |
| - Fixed-effect | 1.40 (1.24-1.58) | 10 |
| - Random-effects | 1.42 (1.21-1.66) | 10 |
| **Maternal exposure pre-pregnancy vs unexposed women** |  |  |
| Danish data sources |  |  |
| *With Gidaya 2014* |  |  |
| - Fixed-effect | 1.56 (1.38-1.77) | 7 |
| - Random-effects | 1.55 (1.32-1.83) | 7 |
| **Maternal exposure during pregnancy vs unexposed women with a history of affective disorder** |  |  |
| Danish data sources |  |  |
| *With Sorensen 2013* |  |  |
| - Fixed-effect | 1.21 (0.98-1.48) | 6 |
| - Random-effects | 1.32 (0.95-1.83) | 6 |
| Swedish data sources |  |  |
| *With Viktorin 2017* |  |  |
| - Fixed-effect | 1.40 (1.25-1.58) | 6 |
| - Random-effects | 1.41 (1.19-1.68) | 6 |

*Only relevant to studies of ASD

**Table S5. Adjusted effect estimates for the different comparator groups in included studies measuring the association between antidepressant exposure and risk of ASD and ADHD substituting estimates for SSRI exposure with any antidepressant exposure.**

|  | Autism spectrum disorder | | Attention Deficit Hyperactivity Disorder | |
| --- | --- | --- | --- | --- |
| **Comparison** | **Adjusted RR** | **No.**  **studies** | **Adjusted RR** | **No.**  **studies** |
| Maternal exposure during pregnancy vs unexposed women | 1.52 (1.30-1.78) | 10 | 1.42 (1.17-1.73) | 7 |
| Maternal exposure pre-pregnancy vs unexposed women | 1.47 (1.27-1.70) | 7 | 1.55 (1.25-1.94) | 5 |
| Maternal exposure during pregnancy vs unexposed women with a history of affective disorder | 1.18 (0.91-1.52) | 6 | 0.98 (0.77-1.24) | 1 |
| Sibling design | 0.95 (0.69-1.31) | 3 | 0.93 (0.75-1.15) | 3 |
| Paternal exposure during the maternal pregnancy period vs. unexposed women | 1.16 (0.99-1.37) | 2 | 1.71 (1.31-2.23) | 1 |

Pooled effect estimates are presented when reported by two or more studies.

**Table S6. Adjusted effect estimates for the different comparator groups in included studies measuring the association between antidepressant exposure and risk of ASD, by study design.**

|  | Autism spectrum disorder | | | |
| --- | --- | --- | --- | --- |
|  | **Cohort** | | **Case control** | |
| **Comparison** | **Adjusted RR** | **No.**  **studies** | **Adjusted RR** | **No.**  **studies** |
| Maternal exposure during pregnancy vs unexposed women |  |  |  |  |
| - Fixed-effect | 1.61 (1.43-1.82) | 6 | 1.55 (1.29-1.86) | 6 |
| - Random-effects | 1.61 (1.37-1.89) |  | 1.47 (1.12-1.93) |  |
| Maternal exposure pre-pregnancy vs unexposed women |  |  |  |  |
| - Fixed-effect | 1.43 (1.25-1.65) | 4 | 1.70 (1.43-2.02) | 4 |
| - Random-effects | 1.43 (1.15-1.78) |  | 1.70 (1.43-2.02) |  |
| Maternal exposure during pregnancy vs unexposed women with a history of affective disorder |  |  |  |  |
| - Fixed-effect | 1.05 (0.85-1.30) | 3 | 1.86 (0.76-4.57) | 1 |
| - Random-effects | 1.12 (0.80-1.57) |  | 1.86 (0.76-4.57) |  |
| Paternal exposure during the maternal pregnancy period vs. unexposed women |  |  |  |  |
| - Fixed-effect | 1.29 (1.08-1.53) | 2 | - | - |
| - Random-effects | 1.29 (1.08-1.53) |  | - |  |

*Sibling studies are a separate design hence not included in this table.

**Table S7. Adjusted effect estimates for the different comparator groups in included studies measuring the association between antidepressant exposure and risk of ADHD, by study design.**

|  | Attention Deficit Hyperactivity Disorder | | | |
| --- | --- | --- | --- | --- |
|  | **Cohort** | | **Case control** | |
| **Comparison** | **Adjusted RR** | **No.**  **studies** | **Adjusted RR** | **No.**  **studies** |
| Maternal exposure during pregnancy vs unexposed women |  |  |  |  |
| - Fixed-effect | 1.48 (1.32-1.66) | 4 | 1.31 (0.99-1.75) | 3 |
| - Random-effects | 1.44 (1.13-1.83) |  | 1.22 (0.76-1.95) |  |
| Maternal exposure pre-pregnancy vs unexposed women |  |  |  |  |
| - Fixed-effect | 1.53 (1.19-1.98) | 2 | 1.28 (1.02-1.60) | 3 |
| - Random-effects | 1.52 (0.96-2.43) |  | 1.28 (1.02-1.60) |  |
| Maternal exposure during pregnancy vs unexposed women with a history of affective disorder |  |  |  |  |
| - Fixed-effect | 0.98 (0.77-1.24) | 1 | - | - |
| - Random-effects | 0.98 (0.77-1.24) |  | - |  |
| Paternal exposure during the maternal pregnancy period vs. unexposed women |  |  |  |  |
| - Fixed-effect | 1.71 (1.31-2.31) | 1 | - | - |
| - Random-effects | 1.71 (1.31-2.31) |  | - |  |

*Sibling studies are a separate design hence not included in this table.

**Table S8. Risk of bias among primary studies measuring the association between maternal antidepressant exposure during pregnancy and risk of ASD in offspring.**

|  | Boukhis  2015 | Brown  2017 | Castro  2016 | Clements  2015 | Croen  2011 | El Marroun  2014 | Eriksson  2012 | Gidaya  2014 | Harrington  2014 | Hviid  2013 | Malm  2016 | Rai  2013 | Sorensen  2013 | Sujan  2017 | Viktorin  2017 |
| --- | --- | --- | --- | --- | --- | --- | --- | --- | --- | --- | --- | --- | --- | --- | --- |
| Reference | 16 | 23 | 17 | 18 | 24 | 19 | 25 | 26 | 27 | 28 | 29 | 30 | 31 | 32 | 33 |
| Baseline and time-varying  confounding | Moderate | Moderate | Moderate | Moderate | Moderate | Serious | Serious | Moderate | Moderate | Moderate | Moderate | Moderate | Moderate | Moderate | Moderate |
| Bias in selection of  participants  into the study | Low | Low | Low | Low | Low | Low | Low | Low | Low | Low | Low | Low | Low | Low | Low |
| Bias in classification of  interventions | Low | Low | Low | Low | Low | Serious | Low | Low | Serious | Low | Low | Serious | Low | Low | Low |
| Bias due to departure  from intended interventions | Moderate | Moderate | Moderate | Moderate | Moderate | Moderate | Moderate | Moderate | Serious | Moderate | Moderate | Moderate | Moderate | Moderate | Moderate |
| Bias due to missing data | Low | Low | Moderate | Moderate | Moderate | Low | Moderate | Moderate | Moderate | Low | Moderate | Moderate | Moderate | Low | Moderate |
| Bias in measurement  of outcomes | Low | Low | Low | Low | Low | Low | Low | Low | Low | Low | Low | Low | Low | Low | Low |
| Bias in selection of the  reported result | Low | Low | Low | Low | Low | Low | Low | Low | Low | Low | Low | Low | Low | Low | Low |
| Overall risk of bias | Moderate | Moderate | Moderate | Moderate | Moderate | Serious | Serious | Moderate | Serious | Moderate | Moderate | Serious | Moderate | Moderate | Moderate |

Assessment conducted using the Cochrane collaboration ROBINS-I tool for assessing risk of bias. Assessment was based upon the risk of bias with the principle analysis comparing the risk of ASD in women who were exposed vs all unexposed women.

**Table S9. Risk of bias among primary studies measuring the association between maternal antidepressant exposure during pregnancy and risk of ADHD in offspring.**

|  | Castro  2016 | Clements  2015 | Figueroa 2010 | Lausegen 2013 | Malm  2016 | Man 2017 | Sujan 2017 |
| --- | --- | --- | --- | --- | --- | --- | --- |
| Reference | 17 | 18 | 34 | 35 | 29 | 36 | 32 |
| Baseline and time-varying confounding | Moderate | Moderate | Moderate | Moderate | Moderate | Moderate | Moderate |
| Bias in selection of participants into the study | Low | Low | Low | Low | Low | Low | Low |
| Bias in classification of interventions | Low | Low | Low | Low | Low | Low | Low |
| Bias due to departures from intended interventions | Moderate | Moderate | Moderate | Moderate | Moderate | Moderate | Moderate |
| Bias due to missing data | Moderate | Moderate | Moderate | Low | Moderate | Low | Low |
| Bias in measurement of outcomes | Low | Low | Low | Low | Low | Low | Low |
| Bias in selection of the reported result | Low | Low | Low | Low | Low | Low | Low |
| Overall risk of bias | Moderate | Moderate | Moderate | Moderate | Moderate | Moderate | Moderate |

Assessment conducted using the Cochrane collaboration ROBINS-I tool for assessing risk of bias. Assessment was based upon the risk of bias with the principle analysis comparing the risk of ADHD in women who were exposed and unexposed.
