# Supplementary figures and images for: Antidepressant use during pregnancy and risk of autism spectrum disorder and attention deficit hyperactivity disorder: systematic review of observational studies and methodological considerations
Source: BMC Med. 2018 Jan 15;16:6. doi: 10.1186/s12916-017-0993-3 (PMC5767968; doi:10.1186/s12916-017-0993-3)

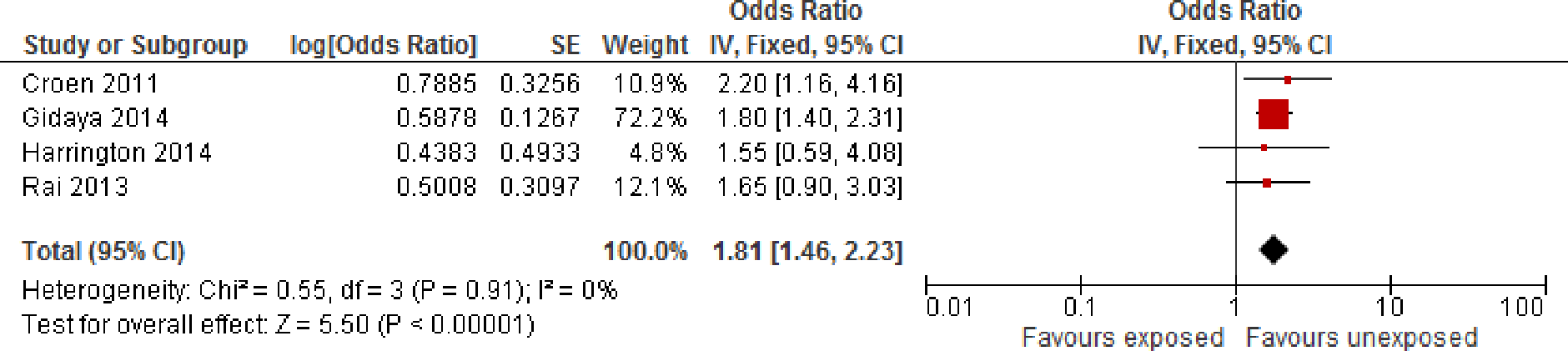

Supplement: Supplementary file 2 — Replication of study results from the previous meta-analysis by Man et al. [15]. (TIF 120 kb) [file 12916_2017_993_MOESM2_ESM.tif]

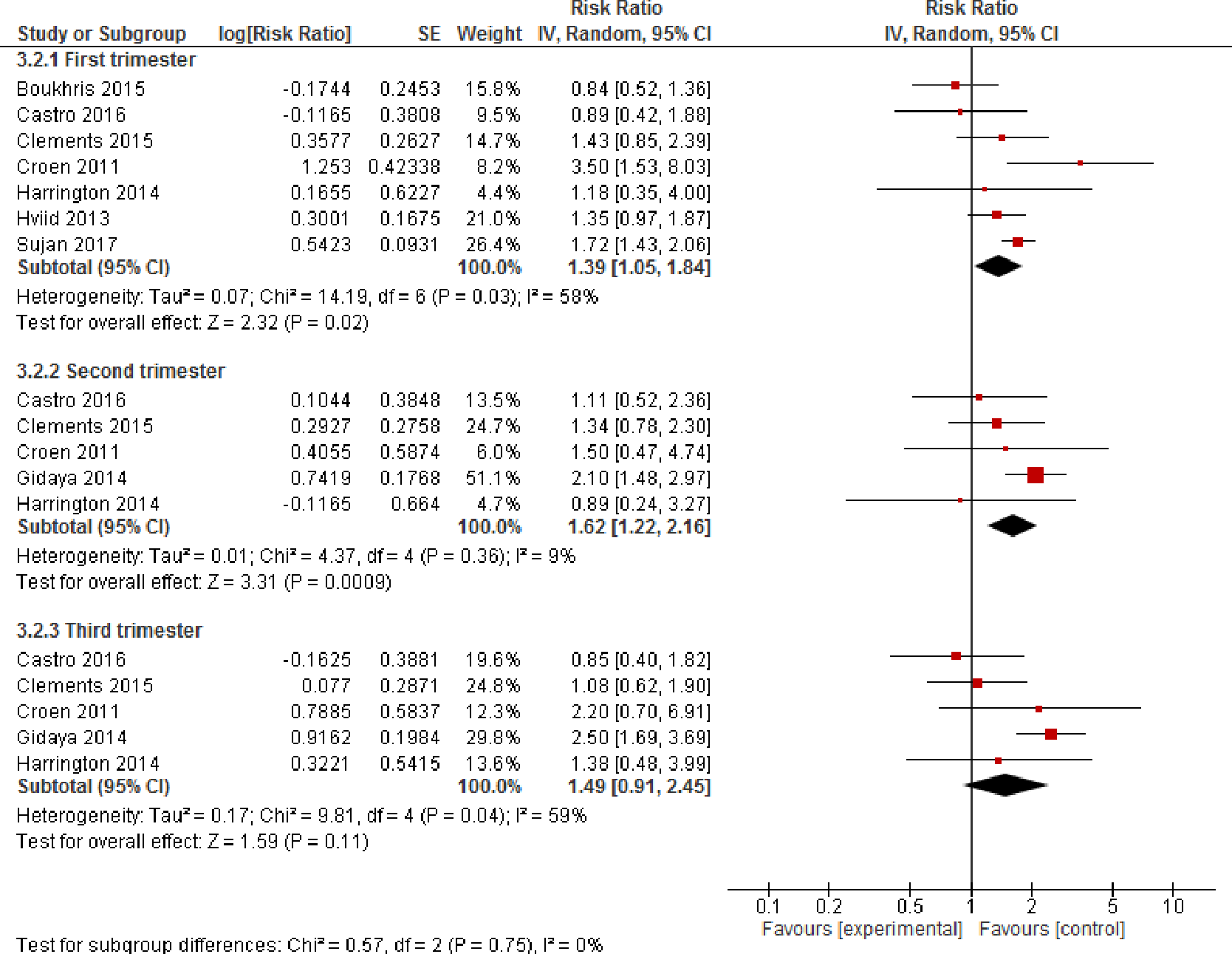

Supplement: Supplementary file 3 — Adjusted effect estimates for the risk of ASD associated with maternal antidepressant exposure during pregnancy compared to unexposed women by trimester. (TIF 304 kb) [file 12916_2017_993_MOESM3_ESM.tif]

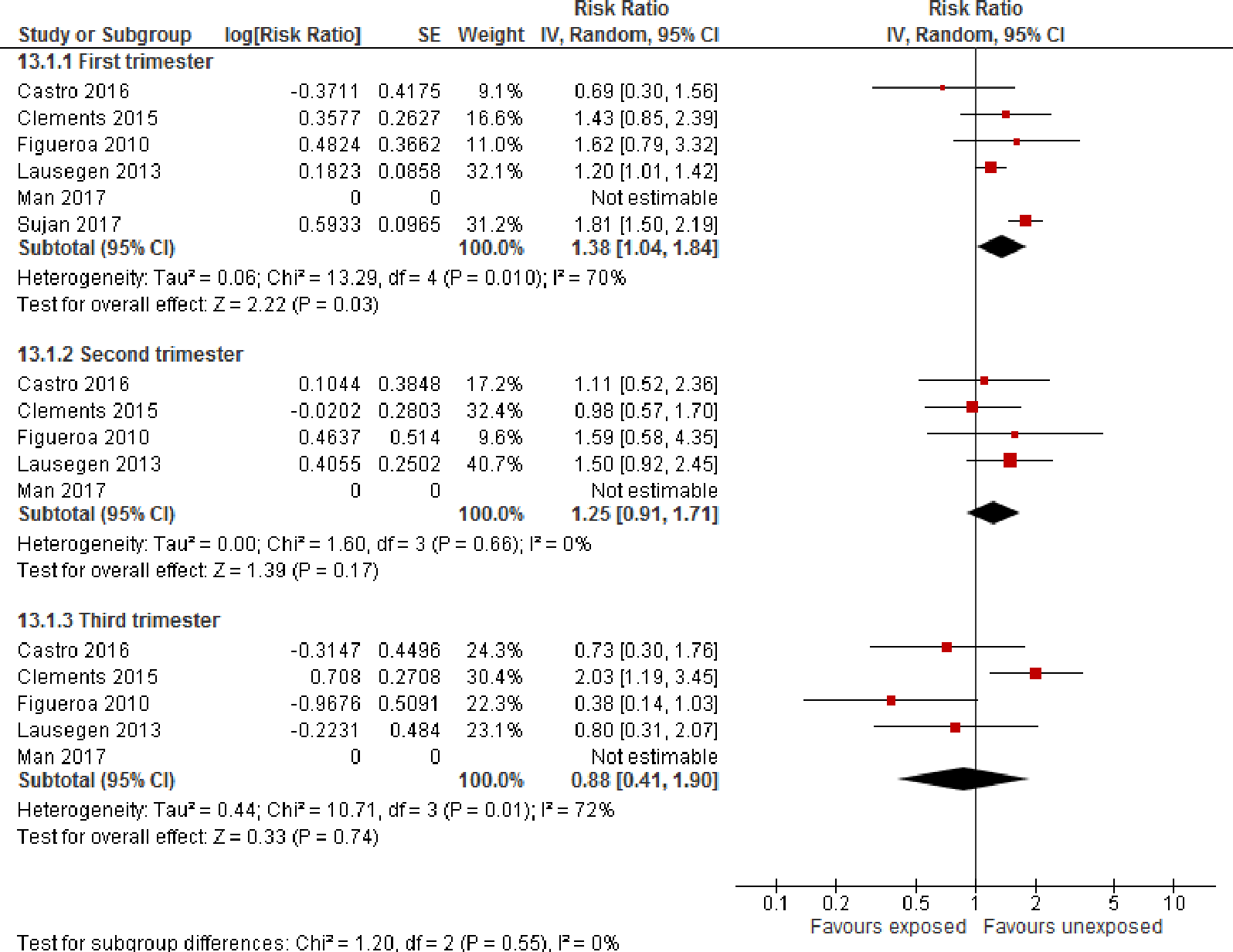

Supplement: Supplementary file 4 — Adjusted effect estimates for the risk of ADHD associated with maternal antidepressant exposure during pregnancy compared to unexposed women by trimester. (TIF 285 kb) [file 12916_2017_993_MOESM4_ESM.tif]

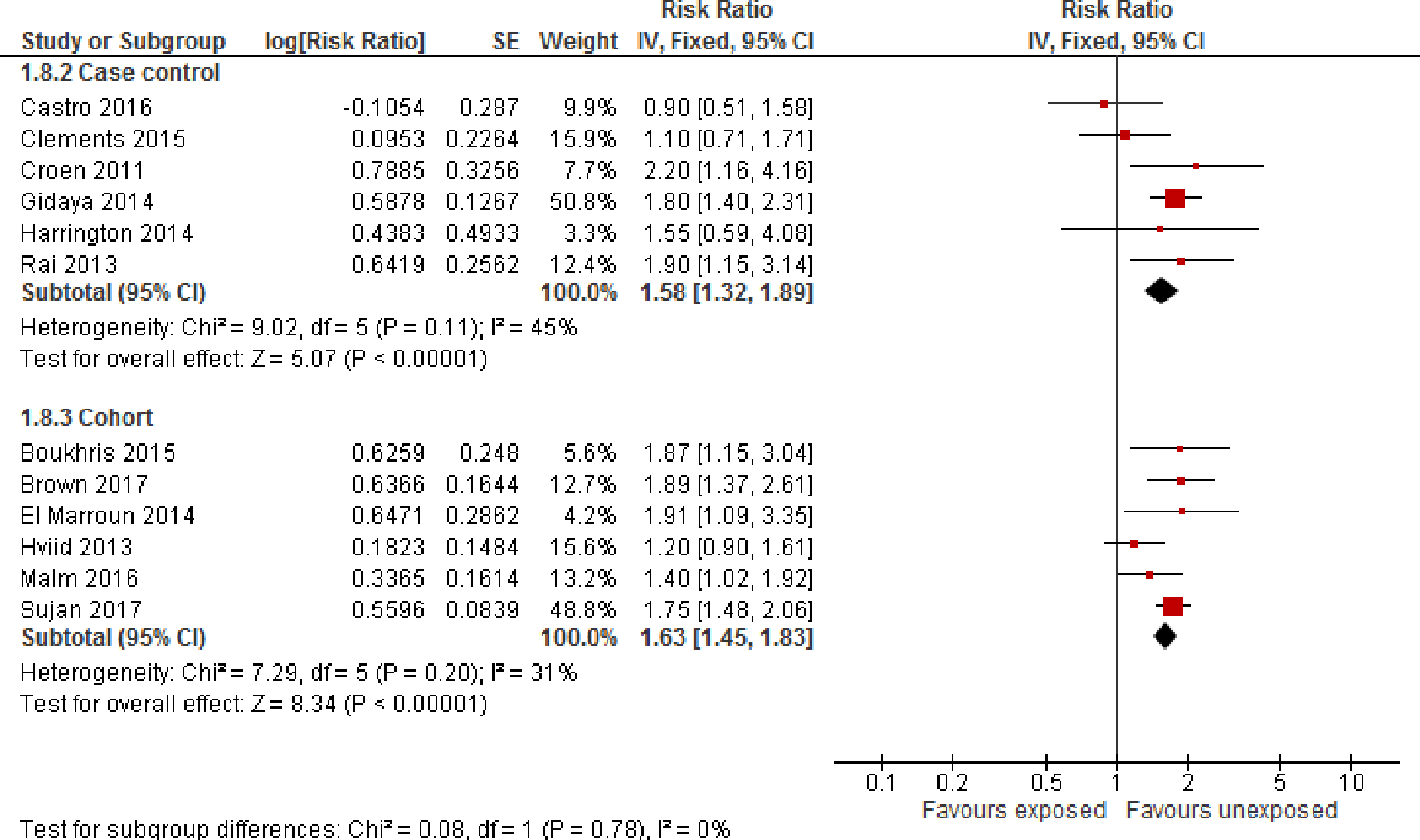

Supplement: Supplementary file 5 — Adjusted effect estimates for the risk of ASD associated with maternal antidepressant exposure during pregnancy compared to unexposed women according to study design. (TIF 274 kb) [file 12916_2017_993_MOESM5_ESM.tif]

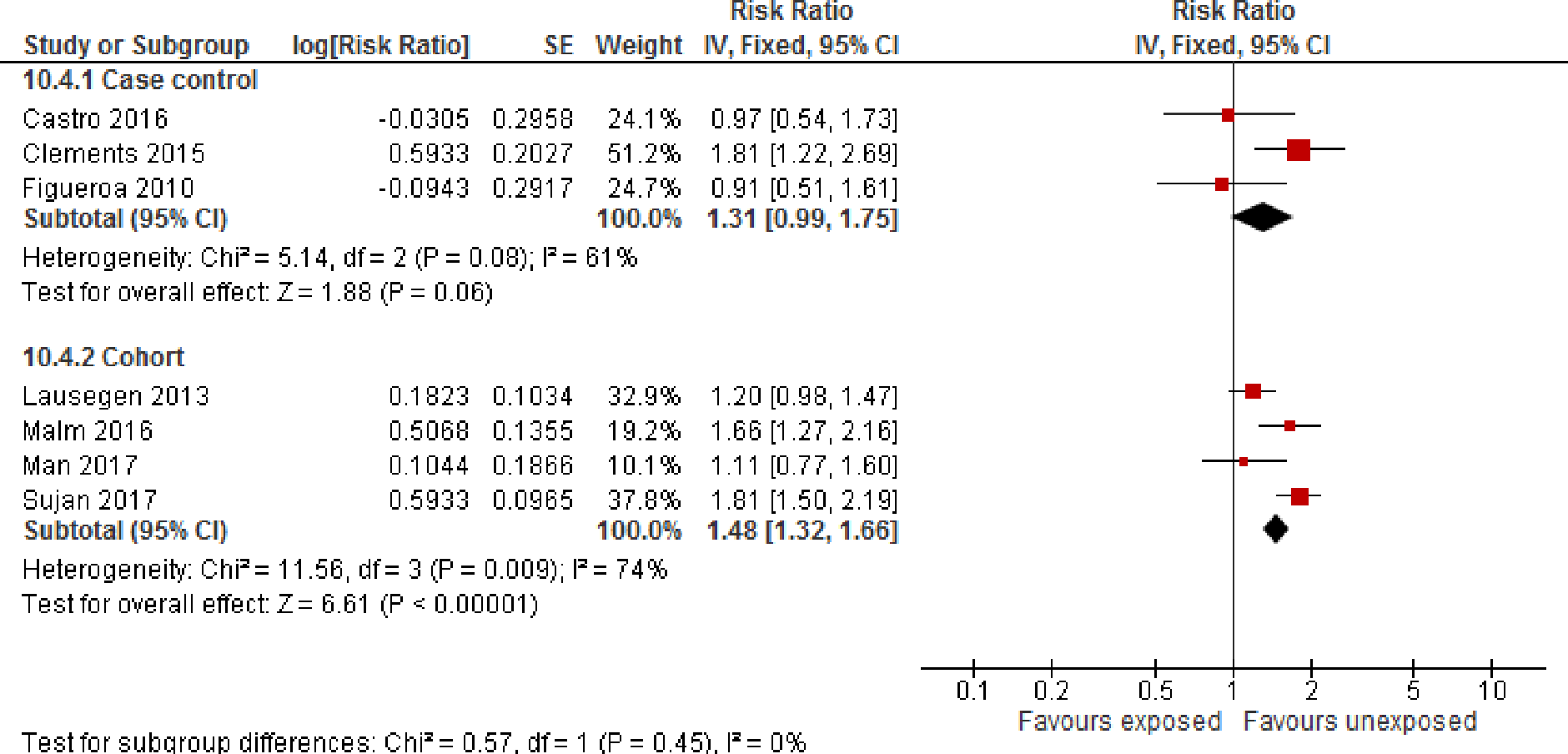

Supplement: Supplementary file 6 — Adjusted effect estimates for the risk of ADHD associated with maternal antidepressant exposure during pregnancy compared to unexposed women according to study design. (TIF 229 kb) [file 12916_2017_993_MOESM6_ESM.tif]

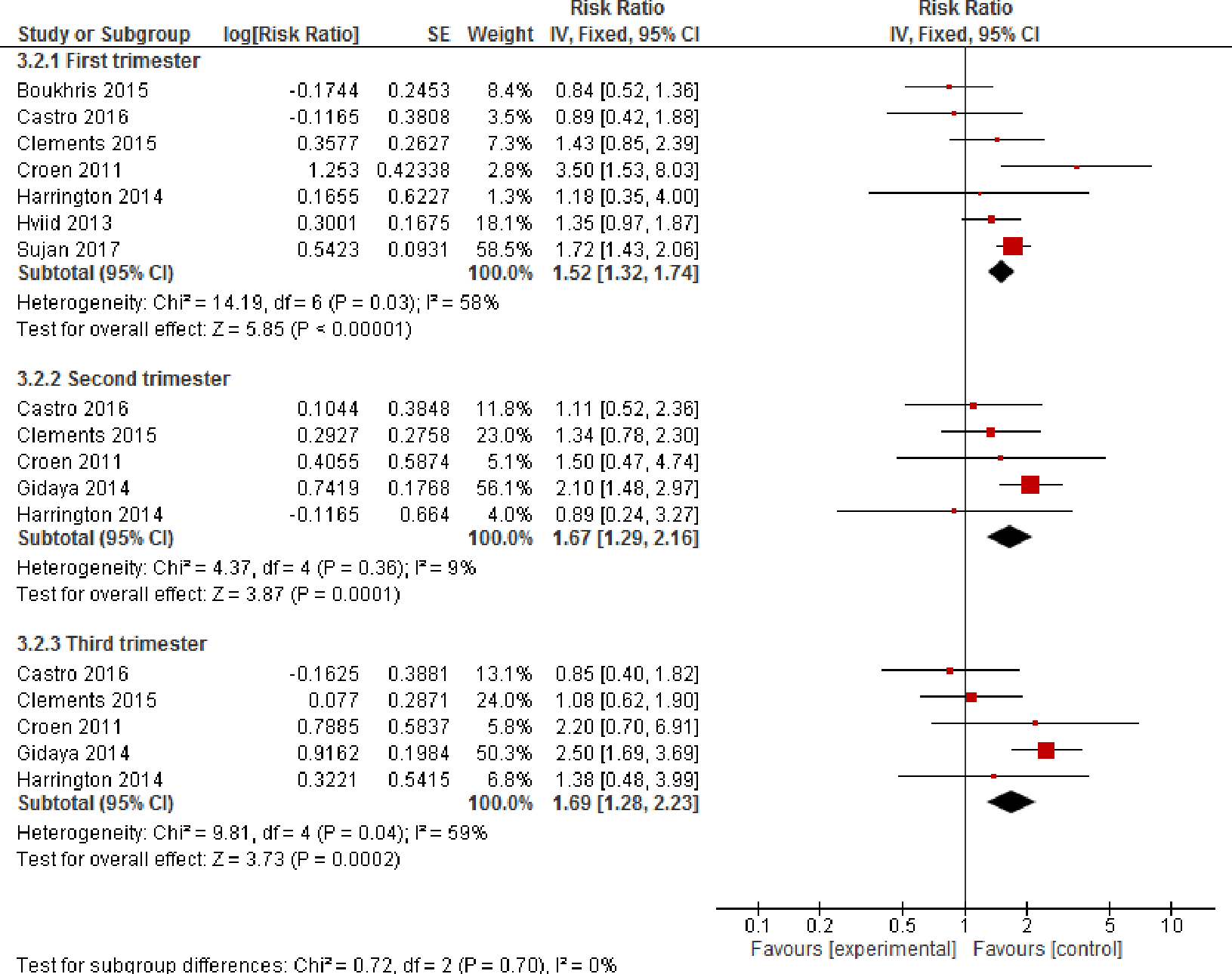

Supplement: Supplementary file 7 — Adjusted effect estimates for the risk of ASD associated with maternal antidepressant exposure during pregnancy compared to unexposed women by trimester (fixed-effect analysis). (TIF 301 kb) [file 12916_2017_993_MOESM7_ESM.tif]

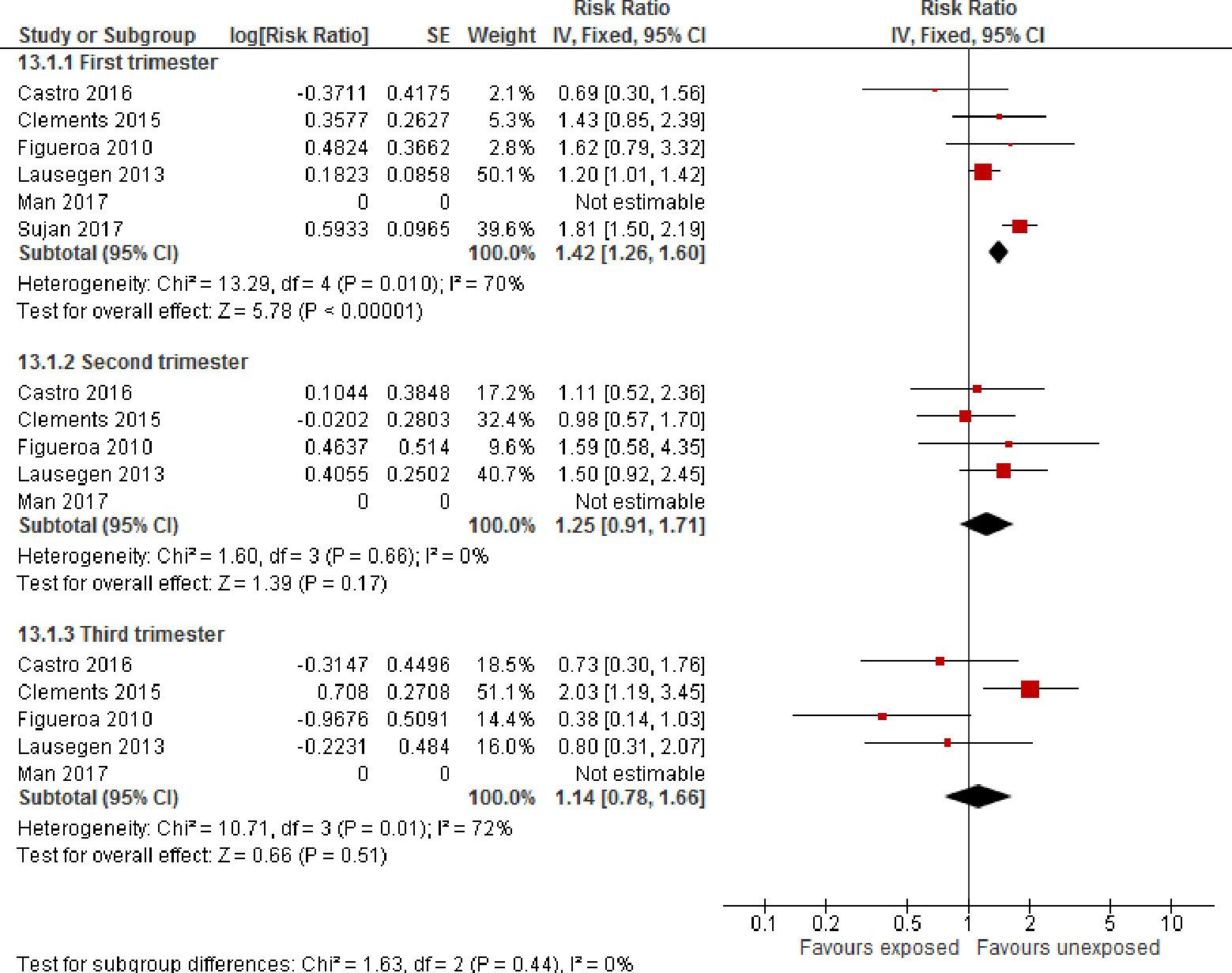

Supplement: Supplementary file 8 — Adjusted effect estimates for the risk of ADHD associated with maternal antidepressant exposure during pregnancy compared to unexposed women by trimester (fixed-effect analysis). (TIF 281 kb) [file 12916_2017_993_MOESM8_ESM.tif]
